# Integrin β_3_ Crosstalk with VEGFR Accommodating Tyrosine Phosphorylation as a Regulatory Switch

**DOI:** 10.1371/journal.pone.0031071

**Published:** 2012-02-17

**Authors:** Xiaoxia Z. West, Nahum Meller, Nikolay L. Malinin, Lalit Deshmukh, Julia Meller, Ganapati H. Mahabeleshwar, Malory E. Weber, Bethany A. Kerr, Olga Vinogradova, Tatiana V. Byzova

**Affiliations:** 1 Department of Molecular Cardiology, Lerner Research Institute, Cleveland Clinic, Cleveland, Ohio, United States of America; 2 Department of Pharmaceutical Sciences, School of Pharmacy, University of Connecticut, Storrs, Connecticut, United States of America; 3 Laboratory of Chemical Physics, National Institute of Diabetes and Digestive and Kidney Diseases, National Institutes of Health, Bethesda, Maryland, United States of America; 4 University Hospitals Harrington-McLaughlin Heart & Vascular Institute and Case Cardiovascular Research Institute, Case Western Reserve University School of Medicine, Cleveland, Ohio, United States of America; University College London, United Kingdom

## Abstract

Integrins mediate cell adhesion, migration, and survival by connecting intracellular machinery with the surrounding extracellular matrix. Previous studies demonstrated the importance of the interaction between β_3_ integrin and VEGF type 2 receptor (VEGFR2) in VEGF-induced angiogenesis. Here we present *in vitro* evidence of the direct association between the cytoplasmic tails (CTs) of β_3_ and VEGFR2. Specifically, the membrane-proximal motif around ^801^YLSI in VEGFR2 mediates its binding to non-phosphorylated β_3_CT, accommodating an α-helical turn in integrin bound conformation. We also show that Y^747^ phosphorylation of β_3_ enhances the above interaction. To demonstrate the importance of β_3_ phosphorylation in endothelial cell functions, we synthesized β_3_CT-mimicking Y^747^ phosphorylated and unphosphorylated membrane permeable peptides. We show that a peptide containing phospho-Y^747^ but not F^747^ significantly inhibits VEGF-induced signaling and angiogenesis. Moreover, phospho-Y^747^ peptide exhibits inhibitory effect only in WT but not in β_3_ integrin knock-out or β_3_ integrin knock-in cells expressing β_3_ with two tyrosines substituted for phenylalanines, demonstrating its specificity. Importantly, these peptides have no effect on fibroblast growth factor receptor signaling. Collectively these data provide novel mechanistic insights into phosphorylation dependent cross-talk between integrin and VEGFR2.

## Introduction

Integrins are a family of transmembrane, heterodimeric glycoproteins composed of alpha and beta subunits. Each integrin subunit contains a large extracellular ligand-binding portion, a single membrane-spanning region, and a short cytoplasmic tail devoid of any enzymatic activity [1]. A crucial characteristic of all integrins, and particularly of the two members of the β_3_ subfamily, α_IIb_β_3_ and α_v_β_3_, is their ability to become activated upon cell stimulation due to agonists or growth factors, a process termed ‘*inside-out*’ signaling. Activated integrins can bind to extracellular matrix components with high affinity and mediate, through ‘*outside-in*’ signaling events, many vital cellular processes such as adhesion, migration, and proliferation [2].

The β subunits' CTs include two tyrosine phosphorylation sites, located within NPxY and/or NPxY-like motifs, and are known to interact with phosphotyrosine binding (PTB) domains of intracellular signaling mediators [3]. These interactions can regulate integrin activation states differentially. For example, talin serves as a major activator for non-phosphorylated β_3_ [4], [5], while Dok1 binds to β_3_ phosphorylated at Y^747^ with a higher affinity and thus, by replacing talin, favors the latent state of the receptor [6]. For α_IIb_β_3_, the biochemical studies suggest that phosphorylation of both tyrosines (Y^747^ and Y^759^) is required for the recruitment of myosin, while phosphorylation of Y^759^ alone is sufficient for interaction with the adapter protein Shc [7], [8]. Our recent structural investigation established the underlying molecular mechanism behind the interaction between the Shc PTB domain and tyrosines phosphorylated (Y^747^, Y^759^) on the β_3_CT [9]. In addition, several studies have demonstrated that tyrosine phosphorylation of β_3_ is involved in the regulation of α_V_β_3_ integrin-dependent adhesion, specifically under conditions of cell activation, and that Y^747^F mutation diminished the stimulation-induced cell adhesion to vitronectin (VN), suggesting that phosphorylation is necessary for a fully functional α_v_β_3_ [10], [11], [12], [13]. In addition, increases in the strength of the α_v_β_3_-VN interaction were shown to be dependent on phosphorylation of β_3_CT [11]. In contrast, α_v_β_3_ mediated adhesion to fibronectin was shown to be abolished with increased β_3_ phosphorylation [14]. Thus, several studies have demonstrated that tyrosine phosphorylation of β_3_ integrin might support different aspects of integrin function. Consequences of integrin phosphorylation might be further modulated by interactions between integrin and intracellular mediators, the presence of which depends upon the cell type and the stage of cell adhesion and spreading.

We have previously demonstrated that α_v_β_3_ function on the endothelium depends on its cross-talk with VEGF type receptor (VEGFR2) [15]. Endothelial cell (EC) stimulation by VEGF promotes a complex formation between α_v_β_3_ and VEGFR2, as well as a conformational change of α_v_β_3_ to a high affinity state [16]. VEGF treatment triggers phosphorylation of β_3_CT on Y^747^ and Y^759^, which are located within the NPxY and NxxY motifs, respectively [16], [17]. Mutations of these residues to phenylalanines inhibit the complex formation between VEGFR2 and β_3_ integrin and VEGF-induced angiogenic responses [16], [17], [18]. The relationship between VEGFR2 and β_3_ appears to be of a reciprocal nature, as β_3_ phosphorylation also leads to enhanced phosphorylation and activation of VEGFR2 [16]. In the presence of normal β_3_ expression, the interplay between the two receptors regulates a number of cellular responses underlying angiogenesis, including EC adhesion, migration, and formation of endothelial tube networks [19], [20]. Based on our and others' findings that Y^747^F-Y^759^F substitutions diminish adhesion to VN and integrin activity, we suggest that phosphorylation of these residues must play a pivotal role in regulation of integrin function [10], [11], [12], [13], [16]. In this study we have defined one of the VEGFR2 binding to β_3_ motifs corresponding to its membrane-proximal region, we have shown that tyrosine-phosphorylation promotes the above interaction between β_3_ and VEGFR2 CTs, and we have proved physiological significance of this interaction by confirming that β_3_ derived Y^747^ containing inhibitory peptide diminishes EC tube formation and angiogenesis. Overall, these data provide novel molecular and mechanistic insights into phosphorylation dependent cross-talk between integrins and VEGF receptors.

## Results

### VEGFR2 interacts with β_3_CT in a phosphorylation dependent manner

**Figure 1:**
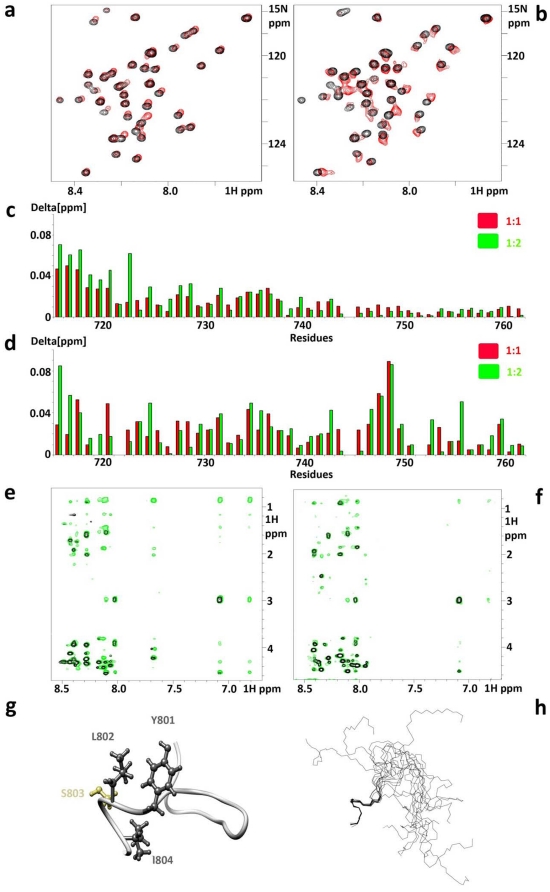
Summary of the *in vitro* evidence for a direct interaction between VEGFR2 and β_3_CTs and structure of the VEGFR2 ^801^YLSI motif in bound conformation. Chemical shift titrations were performed in water at 25°C at pH 6.1 with β_3_ concentrations of in a range of 30–100 µM. Expanded region of ^15^N HSQC spectra show chemical shift perturbations for **a**) β_3_NP. **b**) β_3_MP in presence of VpepA at the ratio 1∶1. **c**) Chemical shift changes in ^15^N labeled β_3_NP upon addition of VpepA at the ratios of 1∶1 (red) and 1∶2 (green). **d**) Chemical shift changes in ^15^N labeled β_3_MP upon addition of VpepA at the ratios of 1∶1 (red) and 1∶2 (green). Delta [ppm] refers to the combined HN and N chemical shift changes according to the equation: Δδ(HN,N) = ((Δδ_HN_^2^+0.2(Δδ_N_)^2^)^1/2^, where Δδ = δ_bound_-δ_free_. Transferred NOEs: all the NOESY experiments were performed in 50 mM NaCl and 25 mM Na-phosphate buffer at pH 6.1 and 25°C with peptide to protein ratio of 50 to 1 and peptide concentrations of 1 mM; **e**) VpepB alone is shown in black and VpepB in combination with GST-β_3_ is shown in green; **f**) VpepC alone (black) and VpepC in combination with GST-β_3_ (green). **g**) Ribbon representation of VpepB structure. Hydrophobic residues of ^801^YLSI region are shown in dark gray. **h**) Backbone superimposition of the 15 lowest energy conformers of VpepB. Residues used for superimposition are ^801^YLSI. Molecular graphics images were produced using the UCSF Chimera package (Pettersen et al., 2004).

Based on our findings that VEGFR2 regulates integrin activation and signaling [21] and that β_3_ tyrosine phosphorylation is crucial for VEGF-induced tyrosine phosphorylation of VEGFR2 [22], we assessed whether the cytoplasmic portion of VEGFR2 binds directly to β_3_CT and how tyrosine phosphorylation affects this interaction. Unlabeled synthetic peptide VpepA (sequence shown in Materials and Methods), representing the thirty membrane proximal residues of VEGFR2 cytoplasmic domain, was titrated into the solution of ^15^N-labeled β_3_NP (non-phosphorylated CT) and ^15^N-labeled β_3_MP (mono-Y^747^-phosphorylated CT). Associated chemical shift perturbations were monitored (Figure 1a and 1b). Chemical shift changes, plotted as a function of the residue number in β_3_CT, are shown in Figure 1c for β_3_NP and Figure 1d for β_3_MP. For non-phosphorylated β_3_CT, maximal perturbations occur near the membrane proximal region (residues ^716^KLLITIHDRK^725^), which might represent the primary binding site for VEGFR2. However, upon phosphorylation, in addition to the above region, maximal perturbations were recorded near the Y^747^ phosphorylation site (^744^NPLYKEA^750^). This finding represents the second binding site and, possibly, increased affinity. Although the chemical shift perturbations were rather modest, they were reproducible and concentration dependent, and reached saturation at a peptide to protein ratio of 3 to 1, indicating the low affinity but specific nature of the observed interaction. Our attempts to calculate the dissociation constants from these titration series were unsuccessful due to low solubility and high tendency of the both VpepA and β_3_CT to precipitate while forming the complex.

Thus, to further confirm and characterize the weak binding of VEGFR2-CTderived peptides to non-phosphorylated β_3_, we have employed an additional trNOE-based approach. β_3_CT was coupled to glutathione S-transferase (GST-β_3_) and two smaller VEGFR2 peptides were synthesized (VpepB and VpepC; see Materials and Methods). TrNOE experiments, the method of choice for studying ultra-weak ligand-receptor pairs [23], were performed and the ratios of peptides to GST-β_3_ were optimized for the most favorable NOE transfer (appeared to happen at a 50 to 1 ratio). The patterns of additional peaks observed for both VpepB and VpepC peptides when mixed with GST-β_3_ were similar (Figure 1e and 1f, respectively), confirming the interaction between peptides and GST-β_3_. However, since more trNOEs were detected for VpepB, this peptide was chosen for further structural analysis. The majority of the additional NOE peaks characterize residues of the region surrounding ^801^YLSI^804^, indicating that this area assumes a bound conformation upon interaction with β_3_NP. It is imperative, however, for the ultra-weak interactions, such as described above, to confirm their specificity. For this reason, we performed negative control experiments. There were no additional peaks in NOESY spectra of GST-VpepB mixtures (data not shown) and thus we can confidently use this system for structural characterization of VEGFR2-derived peptides.

While the structural analysis was reported for extracellular ligand binding [24] and cytoplasmic kinase domains of VEGFR2 [25], no structural information is available regarding the membrane proximal region of VEGFR2. Accordingly, we performed structural calculations for VpepB bound to GST-β_3_. VpepB exhibits a well defined C-terminal region (^801^YLSIV^805^), forming an α-helical turn and an additional loop surrounding two Gly residues (^792^ANGGE^796^), whereas the remaining parts are unstructured. Figure 1g shows a ribbon representation of VpepB along with ball and stick representation for residues ^801^YLSI^804^. Figure 1h illuminates backbone superposition of the 15 lowest energy conformers. Statistics for this ensemble are presented in Table 1 and the sequential connectivity map is shown in Figure S1. The NMR data has been deposited to BMRB (access code 18148).

**Table 1:** Structural statistics for the 15 final NMR structures of VpepB.

**NMR distance constraints**	
Distance constraints	
Total NOE	151
Intra-residue	48
Inter-residue	103
Sequential (|*i*−*j*| = 1)	72
Medium-range (|*i*−*j*<5)	31
Long-range (|*i*−*j*|> = 5)	0
Hydrogen bonds	0
**Structure statistics**	
Violations (mean and s.d.)	
Distance constraints (Å)	0.045+/−0.007
Max. distance constraint violation (Å)	0.366
Deviations from idealized geometry	
Bond lengths (Å)	0.012+/−0.0001
Bond angles (°)	0.75+/−0.039
Impropers (°)	0.36+/−0.027
Average pairwise r.m.s. deviation (Å)^a^	ordered residues 801–804
Heavy	0.5
Backbone	0.1
Ramachandran plot^a^	
Most favored	51.1%
Additionally allowed	44.4%
Generously allowed	4.4%
Disallowed regions	0.0%

a) Ordered residues (residues with sum of phi and psi order parameters <1.8) are considered for r.m.s.d. calculations and Ramachandran statistics.

Collectively, our data demonstrate that non-phosphorylated β_3_CT interacts with VEGFR2, and this interaction involves the membrane proximal region within β_3_CT and ^801^YLSI^804^ motif within VEGFR2. β_3_ Y^747^ phosphorylation generates an additional VEGFR2 binding site within the ^744^NPLYKEA^750^ region of β_3_CT, which is not susceptible to structural characterization using the approach employed for the non-phosphorylated β_3_ (as we were unable to purify GST-fused mono-Y^747^-phosphorylated β_3_CT) and awaits the development of novel strategy for further investigation.

### β_3_CT tyrosine phosphorylation and its consequences in endothelial cells

**Figure 2:**
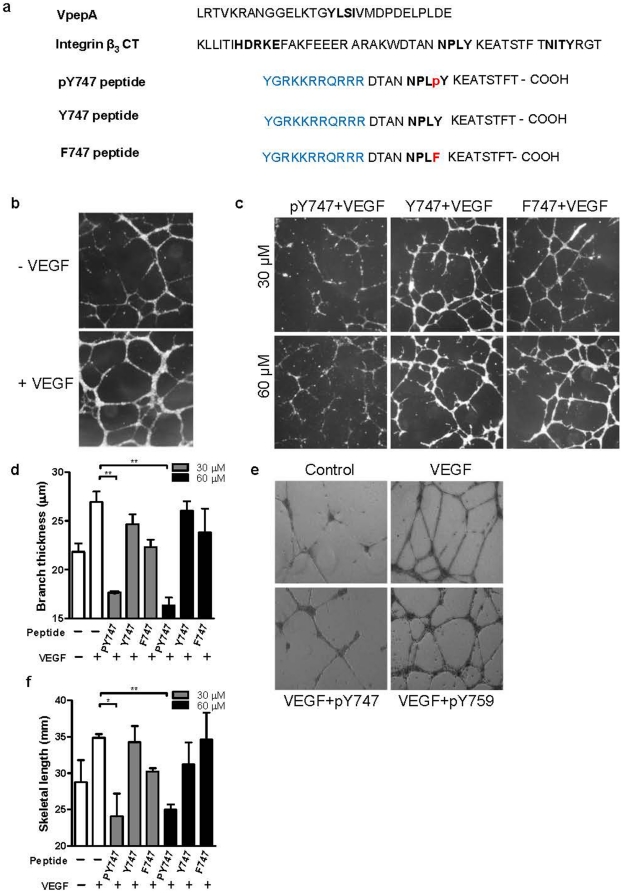
The pY747 peptide inhibits VEGFR2-induced angiogenesis *in vitro*. **a**) The amino acid sequences of VpepA, human integrin β_3_ cytoplasmic tail (CT), and derived peptides. The conserved YLSI (VpepA), HDRKE (Integrin β_3_CT) along with the two tyrosine phosphorylation motifs are shown in orange. HIV-TAT leader sequence (shown in blue) was added to allow delivery of the peptides across the cell membrane. **b**) Representative images of HUVEC tube formation in the presence with or without VEGF (20 ng/mL). **c**) Example images of inhibition of *in vitro* endothelial tube formation by the pY747 peptide. HUVEC were plated on matrigel-coated 48 well plates in the presence of 20 ng/mL VEGF and peptides at indicated concentrations. The cells were allowed to form tubes for 16 hours, bright field images at 2.5× magnification were taken and analyzed (using computer algorithms) for number, length, and thickness of branches. **d**) Quantitative result of branch thickness under different treatments. **e**) Representative images of tube formation in the presence of pY747 and pY759 peptide. **f**) Quantitative result of tube length as indicated.

Since our recent results [26], [27] indicate that phosphorylation of Y^747^ might stabilize the integrin activated state, and the data presented above show that Y^747^ phosphorylation also promotes β_3_CT binding to VEGFR2, we next sought to assess the physiological consequences of β_3_CT phosphorylation in ECs. To this end, we utilized a phosphopeptide containing a 16 amino acid sequence from β_3_CT encompassing the ^744^NPLpY^747^ motif [12] (pY747 peptide; Figure 2a). The peptide is expected to compete with endogenous binding partners for β_3_MP, including the VEGFR2 cytoplasmic domain. Previous studies have utilized the mutations of Y^747^ and Y^759^ to phenylalanines in order to simulate the non-phosphorylated state of β_3_CT. Therefore, in addition to a peptide containing nonphosphorylated ^744^NPLY^747^ motif (Y747 peptide, we used a peptide bearing Y^747^F mutation (F747 peptide) as controls in our studies (Figure 2a). The peptides were conjugated to an HIV-TAT leader sequence to allow delivery into cells. Indeed, the results of confocal microscopy analysis of ECs confirmed the uptake and the presence of the peptides within the cells and, most importantly, on the cell surface (Figure S2).

**Figure 3:**
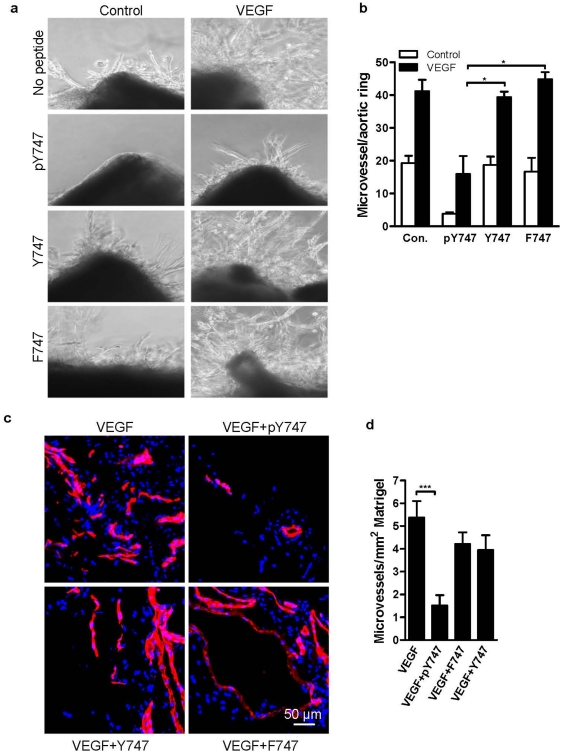
The pY747 peptide inhibits VEGFR2-induced angiogenesis in *ex vivo* and *in vivo*. **a**) Inhibition of *ex vivo* endothelial sprouting by the pY747 peptide. Mouse aortic rings were embedded in matrigel in the presence or absence of 40 ng/mL of VEGF and peptides as indicated. Photographs were taken at three days and the number of endothelial sprouts originating from each ring was determined. **b**) Quantification of aortic ring assay as indicated in Fig. 3a. **c**) Inhibition of *in vivo* angiogenesis by pY747 peptide. Results of matrigel plug angiogenesis assay are shown. The indicated peptides at 200 µM concentration were mixed with growth factor-reduced matrigel containing VEGF (500 ng/mL) and injected subcutaneously into wild type mice. Seven days later, the matrigel implants were removed, sectioned, and blood vessels were stained with CD31 Ab (red) and nuclei with DAPI (blue). Vessel area was determined using ImagePro. **d**) Quantified results of matrigel plug assay as indicated in Fig. 3c.

To assess the requirement of Y^747^ phosphorylation of β_3_ for integrin-dependent angiogenic responses of ECs, three experimental systems were utilized. During angiogenesis, ECs re-organize to form a three-dimensional vessel structure [28], and this could be modeled *in vitro* in tube formation assays. Accordingly, tube formation by HUVEC treated with VEGF was assessed in the presence of β_3_CT-derived pY747, Y747, or F747 peptides. As shown in Figure 2b, VEGF-treated HUVEC formed a robust endothelial network. The tyrosine-phosphorylated peptide (pY747) inhibited this process, resulting in a disruption of the network's structure (Figure 2c). The thickness (Figure 2d), as well as the tube length (Figure 2f), of endothelial branches formed in the presence of pY747 peptide was significantly decreased compared to untreated cells. In contrast, cells treated with control F747 peptide were able to form a network of endothelial cords as robust as seen in untreated cells (Figure 2c). Unphosphorylated Y747 peptide exhibited only a weak or negligible effect (Figure 2c). In a similar assay, a peptide containing pY^759^ sequence from the second NPxY-like motif of β_3_ had no inhibitory effect, emphasizing the unique role of pY^747^ residue (Figure 2e). In the aortic ring assay, pY747 was even more potent. The treatment with pY747, but not F747 or Y747 peptide, inhibited the sprouting of ECs stimulated by VEGF by more than 60% (Figure 3a and 3b). Next, the effect of pY747 peptide on *in vivo* angiogenesis induced by VEGF was evaluated. Matrigel containing (or lacking) VEGF and peptides was injected subcutaneously in C57BL/6 (wild type) mice. Seven days later, matrigel implants were removed, sectioned, and blood vessels were stained using CD31 antibody. The pY747 peptide inhibited VEGF-induced vascularization by more three-fold, while the control F747 or Y747 peptides had no effect (Figure 3c and 3d).

**Figure 4:**
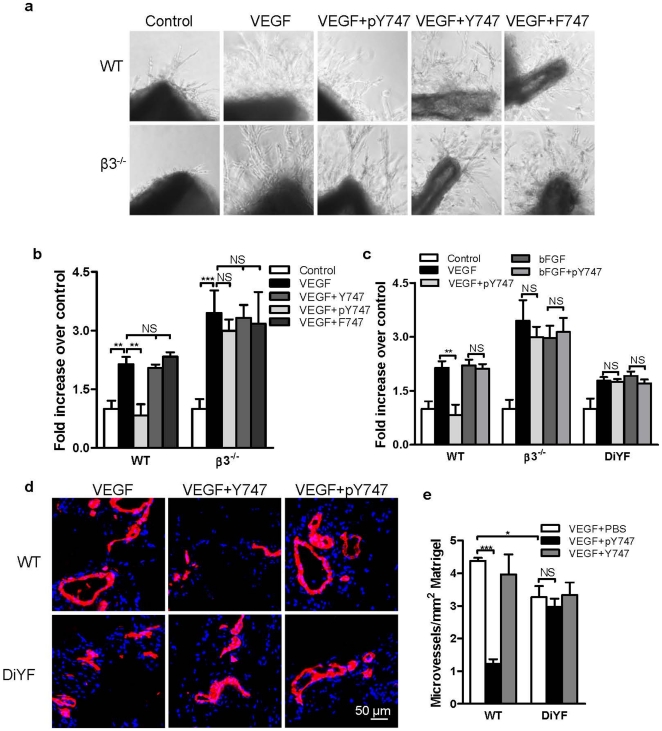
The pY747 peptide has no effect on β3^−/−^ or DiYF mice. **a**) pY747 peptide does not inhibit VEGF-induced aortic ring growth from β3^−/−^ mice. Mouse aortic rings were embedded in matrigel in the presence of 40 ng/mL of VEGF and 40 µM of peptides as indicated. **b**) Quantification of aortic ring assay as indicated in Fig. 4a. **c**) pY747 could not inhibit bFGF-induced aortic ring growth, Mouse aortic rings were isolated from wild type (WT), β3^−/−^, and DiYF mice and embedded in matrigel in the presence of 40 ng/mL of VEGF, 20 ng/mL of bFGF or pY747 peptides as indicated. Aortic rings were incubated for 3 days for wild type and β3^−/−^ aortic rings and 4 days for DiYF aortic rings (longer incubation was used to obtain visible aortic sprouting which is diminished in these mice). **d**) pY747 peptide does not inhibit angiogenesis in DiYF mice. Peptides' effect on *in vivo* angiogenesis in wild type mice and DiYF mice was tested as described. **e**) Quantification of blood vessels in matrigel plus assay as indicated.

The pY747 peptide is predicted to mimic and compete with the phosphorylated form of endogenous β_3_ integrin and, therefore, to also inhibit β_3_ phosphorylation-dependent responses. Thus, this peptide should not have any inhibitory activity in mice characterized by the lack of β_3_ phosphorylation (β_3_ knock-out mice or DiYF knock-in mice [29]), which, in turn, diminishes the complex formation between β_3_ and VEGFR2 [16], [30]. Indeed, as shown in Figure 4a and 4b, pY747 phosphopeptide could not effectively inhibit angiogenesis induced by VEGF in the aortic ring from β3^−/−^ mice, which is corroborated by the experiments with DiYF knock-in mice (Figure 4c). In addition, pY7474 peptide inhibition was specific to VEGF as no inhibition of basic fibroblast growth factor (bFGF) angiogenesis occurred in wild type, β3^−/−^, or DiYF mice (Figure 4c). Furthermore, the results from matrigel plug assay demonstrated that pY747 peptide resulted in inhibition of VEGF-induced angiogenesis in wild type, but not in DiYF mice, demonstrating the specificity of the approach (Figure 4d and 4e). Together, these results indicate that β_3_CT phosphorylation at Y^747^ positively regulates integrin-dependent responses in angiogenesis.

### VEGF-induced VEGFR2 phosphorylation and downstream signaling are diminished by pY747 peptide

**Figure 5:**
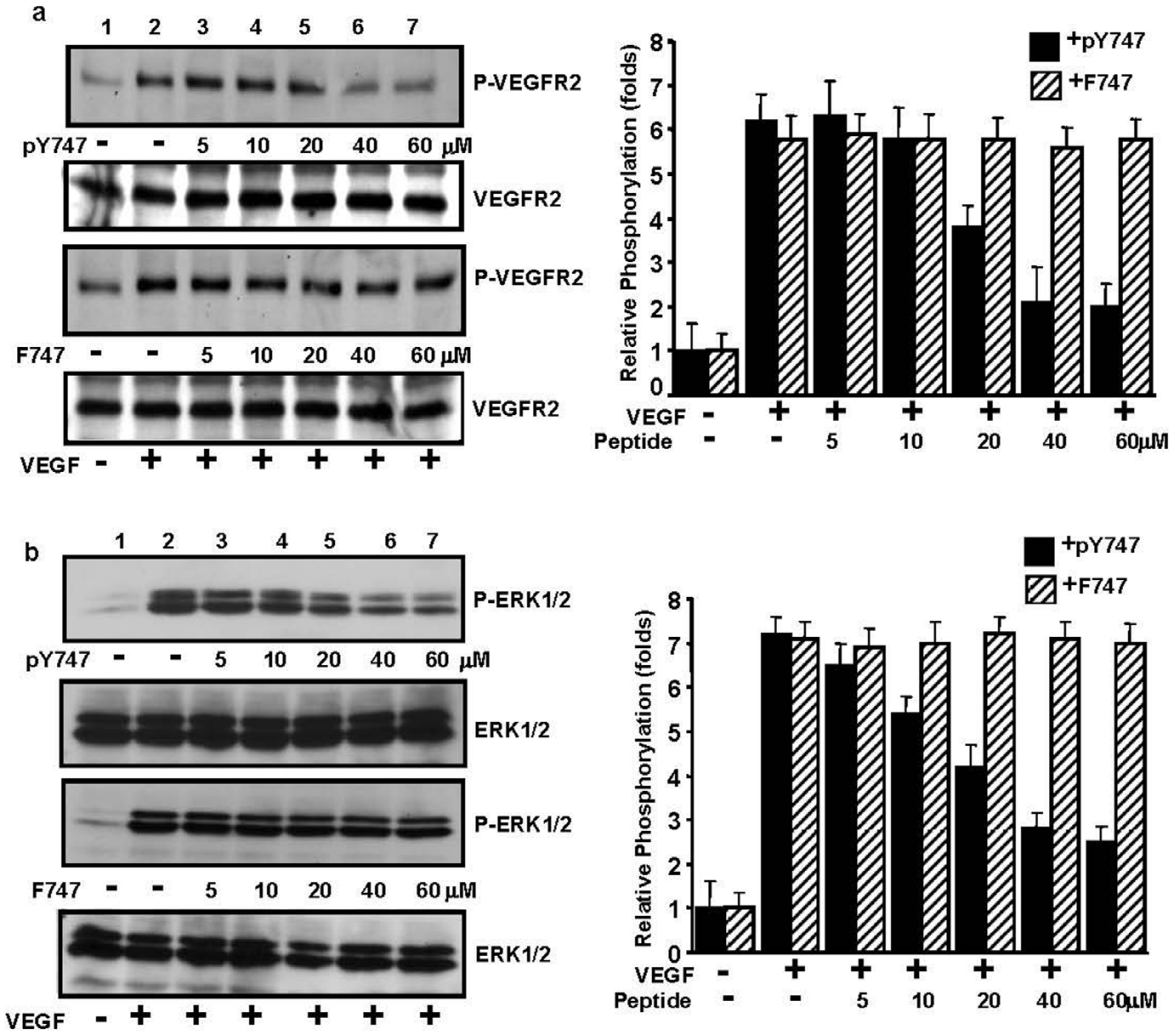
pY747 peptide inhibits VEGF-induced VEGFR2 phosphorylation and ERK activation. Serum starved HUVEC were incubated with the indicated concentrations of pY747 or F747 peptides for 3 h, then stimulated with 20 ng/mL VEGF for five min at 37°C or left unstimulated. The cells were lysed and equal amounts of protein from total cell lysates were subjected to Western blot analysis with **a**) anti-p-VEGFR2 (Y1775) Ab or **b**) anti-p-ERK1/2 antibodies. The blots were reprobed with **a**) anti-total VEGFR2 or **b**) anti-total ERK1/2 antibodies as loading control. Bands were quantified by densitometric analysis and fold increase over unstimulated cells are displayed (right panels).

Studies from our lab and others demonstrated cross-activation of VEGFR2 and α_v_β_3_ [16]. To test whether VEGFR2 activation and subsequent signaling were affected by pY747 peptide, we assessed Y^1175^ phosphorylation of VEGFR2, a major VEGF-dependent VEGFR2 autophosphorylation site implicated in EC migration, along with ERK phosphorylation and subsequent DNA synthesis [31], [32]. To this end, the phosphorylation status of Y^1175^ of VEGFR2 and ERK1/2 was assessed in ECs, which were treated with peptides with or without VEGF stimulation. Western blot analysis shows that treatment of cells with pY747 peptide resulted in inhibition of VEGFR2 and ERK1/2 phosphorylation in a dose-dependent manner with the maximal effect occurring at 40 µM or higher concentrations; the control F747 peptide, however, had no effect (Figure 5). Thus, interference with intracellular signaling mediated by phosphorylated β_3_CT results in impaired activation of VEGFR2 and proangiogenic signaling events downstream of VEGFR2.

In contrast to VEGF, pY747 peptide had no effect on bFGF-induced activation of downstream signaling molecules, represented by phospho-ERK. As shown in Figure S3, ERK activation by bFGF was not affected in the presence of either pY747 or control F747 peptide. In these experiments, effects of VEGF were neutralized by VEGFR inhibitor, AAL-993. Together with results of NMR experiments (Figure 1) and previously published observations [16], [18], these data further emphasize the specificity and importance of a cross-talk between VEGFR2 and β_3_ integrin. Together, our findings provide a structural basis for the cross-talk between β_3_CT and VEGFR2 and show that this cross-talk mediates important cellular processes, such as VEGFR2 activation, EC tube formation, and angiogenesis.

## Discussion

Over the last decade, the importance of the interplay between integrins and growth factor receptors was demonstrated in a number of physiologically important processes, including angiogenesis. At the same time, tyrosine phosphorylation of β_3_ integrin has been identified as a major regulatory event for the modulation of this integrin function and its cross-talk with VEGF receptor. In the presented work, we have focused on the intertwining of these two processes. Using a NMR approach, we have documented a direct interaction between VEGFR2 and β_3_ CTs, which appeared to be dependent upon Y^747^ phosphorylation of β_3_.

In platelets, Y^747^ phosphorylation of β_3_ occurs as a consequence of ligand binding and receptor clustering [33]. In this case, tyrosine phosphorylation appears to be involved in outside-in signaling [7], [29]. In ECs, however, Y^747^ phosphorylation of β_3_ occurs in response to VEGF stimulation in the absence of ligand. Here, we have shown that the phosphopeptide containing pY^747^ acts as an antagonist of VEGF-induced and integrin-mediated responses. It inhibits cellular processes known to be dependent on β_3_ integrin activation, such as VEGF-induced endothelial tube formation, sprouting, and angiogenesis *in vivo*. Importantly, the phosphopeptide containing pY^747^ is a highly specific inhibitor for processes dependent on β_3_ and its phosphorylation, since this peptide has no effect in DiYF knock-in mice expressing mutant β_3_ unable to undergo phosphorylation as well as in β_3_ knock-out mice.

Previous studies using cell systems with “activatable” α_V_β_3_, such as myeloid K562 cells, demonstrated that that substitution of Y^747^ by phenylalanine impaired agonist-induced adhesion to VN [10], [13]. A subsequent study demonstrated that firm adhesion of K562 cells to VN proceeds through three steps, and it is the second step characterized by a four-fold increase in receptor-ligand binding strength that was shown to be dependent on phosphorylation of Y^747^. In ECs, DiYF mutations of both Y^747^ and Y^759^ to phenylalanines affected VEGF-induced activation of α_V_β_3_ and endothelial responses [16]. However, in Chinese hamster ovary (CHO) cells, expressing α_V_β_3_, a cell model generally characterized by the lack of inside-out integrin signaling, the Y^747^F mutation did not affect binding of fibrinogen-coated beads [33]. A possibility of indirect inhibitory effect of β_3_ phosphorylation was reported in a study utilizing expression of temperature-sensitive v-Src in osteosarcoma cells. In this study, increased β_3_ phosphorylation correlated with reduced α_V_β_3_-fibronectin binding strength, which was rescued by the Y^747^F mutation [14]. However, many mutagenesis studies using a well-defined integrin activation monitoring system, i.e. binding of soluble monovalent ligand, demonstrate that mutations of Y^747^ to F generally diminish integrin function. This effect might also be attributed to the disruption of binding sites for key integrin activators, such as talin and kindlin. In this regard, our data showing differential effects of β_3_ phosphopeptide containing pY^747^ versus its unphosphorylated form are crucial in demonstrating the role of phosphorylation of this integrin, *per se*. Of particular interest is the fact that the effect of phosphorylation may differ for other subfamilies of β integrins. For example, in mice whose tyrosines of the β_1_ tail were mutated to phenylalanines (YY^783/795^FF), no obvious defects have been observed [34]. Again, use of the Y to F mutations need to be interpreted with an understanding that these mutations might affect events other than the direct consequences of phosphorylation. These reservations concerning mutagenesis studies emphasize the importance of the structural analysis presented in this study, which allowed direct comparison of phosphorylated versus unphosphorylated form of β_3_.

Y^747^ phosphorylation in β_3_CT appears to promote a complex formation with VEGFR2, and this complex is not present in DiYF mutant cells [18]. Our data show not only the presence of the β_3_-VEGFR2-CTs complex, but also the key regulatory role of the Y^747^ phosphorylation site in this complex formation. Furthermore, β_3_CT-derived Y^747^-phosphorylated peptide was able to disrupt VEGF-induced signaling, EC tube formation, and angiogenesis, in contrast to the unphosphorylated control. Thus, our *in vitro* data supports *in vivo* observations that β_3_CT phosphorylation is crucial for the interaction and cross-talk with VEGFR2 [16], [18]. This interaction performs a key regulatory function in pathological angiogenesis [18], and new structural insights into this mechanism may permit the design of novel anti-angiogenic compounds. While we demonstrate that phosphorylation of the Y^747^ likely increases the affinity of β_3_ for VEGFR2-derived, membrane-proximal peptides, additional phosphorylation of Y^759^ might diminish this effect (data not shown). This finding may indicate a key role for the NPLY^747^ motif in positive regulation of the cross-talk between VEGFR2 and β_3_ [30], which is in agreement with differences in the kinetics of Y^747^ vs. Y^759^ phosphorylation in response to VEGF in ECs [18].

To conclude, we have shown direct complex formation between cytoplasmic tails of β_3_ integrin and VEGFR2 *in vitro*, structurally characterized one of the VEGFR2 binding motifs, and confirmed that the above interaction is further enhanced by Y^747^ phosphorylation of β_3_ integrin. We also showed *in vivo* that pY^747^ affects β_3_ integrin cross-talk with VEGFR2 resulting in suppressed VEGF-induced signaling, endothelial tube formation, and angiogenesis. These findings identify important regulatory elements controlling the activity of β_3_ integrins in ECs, which underlie a number of more complex responses, including thrombosis/haemostasis and pathological angiogenesis.

## Materials and Methods

### Peptide synthesis

Short peptides corresponding to the membrane proximal region of VEGFR2, VpepA (^786^LRTVKRANGGELKTGYLSIVMDPDELPLDE^815^), VpepB (^786^LRTVKRANGGELKTGYLSIV^805^), VpepC (^791^RANGGELKTGYLSIVMDPD^809^), and membrane-permeable corresponding to β_3_CT peptides, pY^747^(YGRKKRRQRRRDTANNPLpYKEATSTFT),Y^747^ (YGRKKRRQRRRDTANNPLYKEATSTFT), and F^747^ (YGRKKRRQRRRDTANNPLFKEATSTFT) were synthesized in the Cleveland Clinic Molecular Biotechnology Core laboratory byFmoc chemistry using solid phase Omega 396 synthesizer (Advanced ChemTech, Louisville, KY). Quality analysis of the peptides was performed by HPLC on an analytical reverse phase C-18 column and by matrix assisted laser desorption ionization time-of-flight (MALDI-TOF) mass spectrometry (MS).

### Expression and Purification

Cloning, expression, and purification of β_3_CT and GST-β_3_ have been described previously [5], [35]. To produce ^15^N isotopically labeled β_3_CT cells were grown in M9 minimal medium containing ^15^NH_4_Cl (1.1 g/L). Tyrosine phosphorylation of β_3_CT has been achieved *in vivo* by using TKB1 bacterial cell line from Stratagene following the manufacture's protocol for the recombinant protein induction as described elsewhere [26].

### NMR Spectroscopy

^1^H-^15^N Heteronuclear single quantum correlation (HSQC) titration experiments were performed in water at 25°C on Varian Inova 600 MHz equipped with inverse-triple resonance cryoprobe. Chemical shifts assignments have been determined previously [5] and have been modified to address the effect of phosphorylation. Transferred NOESY experiments for different peptides were performed at pH 6.1. Different ratios of the peptides to the binding partner were investigated to find the optimal range for NOE transfer for each particular analysis. All the spectra were processed with NMRPipe [36] and analyzed by CCPN software suite [37]. The resonance assignments of unlabeled peptides were made using conventional 2D ^1^H-^1^H TOCSY and NOESY spectra [38] by CCPN software suite [37].

### Structure Calculation

Sequence-specific assignments of integrin tails are described elsewhere [5]. Restraints from two dimensional ^1^H-^1^H NOESY experiment were used for the structure calculations. The structures were calculated based on the hybrid distance geometry-dynamical simulated annealing method using the X-PLOR-NIH [39]. The target function minimized during simulated annealing (as well as during conventional Powell minimization) comprises only quadratic harmonic terms for covalent geometry, square-well quadratic potentials for the experimental distance restraints. Best structures from the ensembles have been chosen based upon lowest Lennard–Jones potential. None of the structures have NOE violations greater than 0.5 Å. The Protein Structure Software suite (PSVS; courtesy of CABM Structural Bioinformatics Laboratory, Rutgers State University of New Jersey) was used for structure validation (http://psvs-1_3.nesg.org/).

### Materials and Animals

Rabbit polyclonal anti-VEGFR2, anti-β_3_-integrin, and mouse monoclonal anti-phospho tyrosine antibodies were purchased from Santa Cruz Biotechnology, Inc. (Santa Cruz, CA). Anti-ERK1/2, Anti-p-ERK1/2, and anti-phospho-VEGFR2 were from Cell Signaling Technology (Beverly, MA). VEGF was purchased from R&D Systems (Minneapolis, MN) and matrigel was obtained from BD Biosciences (San Jose, CA). DiI was obtained from Invitrogen (Carlsbad, CA). Drabkin's reagent was from Sigma. C57BL/6 (wild type), β_3_ knock-out (C57BL/6 background), and DiYF knock-in (in DiYF mice, the β_3_ integrin tyrosines 747 and 759 are mutated to phenylalanine, C57BL/6 background [29]) mice were housed and treated according to Cleveland Clinic Institutional Animal Care and Use Committee regulations.

### Cell culture

Human umbilical cord vein endothelial cells (HUVEC) were grown in DMEM:F12 media supplemented with 15% FBS, 100 U/mL penicillin, 100 µg/mL streptomycin, 90 µg/mL heparin sulfate, and 90 µg/mL endothelial cell growth factor. Lung ECs: Mouse lungs were excised, minced, and digested using a collagenase-dispase reagent (Roche Diagnostics, Indianapolis, IN). Digests were strained and the resulting cell suspension was plated on flasks coated with 10 µg/mL fibronectin in HUVEC growth media.

### Aortic ring, tube formation, and in vivo Matrigel angiogenesis assays

The aortic ring assays were performed as described previously [18].

The formation of vascular tube-like structures by HUVEC was performed as described previously [40] with modification. We coated 48-well plates with 200 µL of growth-factor reduced Matrigel according to the manufacturer's instructions. HUVECs were starved 3 h in DMEM:F12, 1% FBS, and 90 µg/mL heparin. The cells were collected by trypsinization, suspended in starvation media, and 60,000 cells were plated per well in the presence or absence of the indicated peptides or VEGF at the indicated concentrations. 16 h later, the cells were washed 2× in PBS, fixed with 2% formaldehyde, and bright field images of the wells were taken.

Matrigel angiogenesis assays were done as described [41]. Growth factor-reduced matrigel was mixed with 500 ng/mL VEGF and 200 µM of peptides, and 400 µL injected into mice subcutaneously. At seven days, the matrigel implants were surgically removed in OCT freezing medium and 7 µm thick sections were prepared. Sections were fixed with 4% paraformaldehyde, incubated with Rat anti-mouse CD31 (BioLegend), and exposed to anti-rat Alexa Fluor568 (Invitrogen). The slides were mounted with medium (DakoCytomation) and images were taken by a TCS-SP (Leica) microscope. For quantification, the images were analyzed with ImagePro software (Media Cybernetics).

### Immunocytochemistry Analysis

HUVEC were grown in monolayer on glass slides and then treated with fluorescein-labeled peptides for the indicated time periods. The cells were further stained with the lipophilic tracer DiI to stain the cell membrane following the manufacturer's instructions, then fixed with 2% paraformaldehyde for 10 min, washed, mounted with cover slips, and analyzed by confocal microscopy (Leica).

### Tube Formation Analysis

Network analysis of tube forming HUVEC was performed in an automated fashion using customized visual basic macros developed within Image-Pro Plus (v6.2, Media Cybernetics, Silver Spring, MD). Bright-field images were imported into Image-Pro one-by-one, in batch mode. A high-pass spectral filter was applied to each image to enhance/equalize intensity; enabling application of a fixed threshold to segment the cell network (will be referred to as the “tube mask”). Since branches were sometimes thin (1 pixel thick), node to node branches were often disconnected following segmentation, even though visual observation confirmed continuity. To resolve this issue, a second approach was applied to preserve continuity. A top-hat morphological filter was applied to the original image to enhance the appearance of low intensity signal, and segmented using a fixed intensity and area threshold. This image was then “added” to the tube mask and inverted (intensity across the tube, referred as “lumens of each tube,” was converted to 255). Using Euclidean distance map (EDM)-based background clustering, the lumen of each tube was segmented such that no lumen was in contact with another. This image was then inverted and “skeletonized” to create continuous single pixel-width medial lines along the entire tube network. To avoid errors due to the “skeletonization” process, an additional algorithm was applied to cluster nodes that were within a given distance (pre-determined value confirmed visually). These nodes were then classified as 3−, 4−, or 5+ branch nodes and summed for each category for output to Microsoft Excel. In addition to incorrect node classification, the skeletonization process can also produce spurious branches. To eliminate these, a “pruning” filter was applied to remove branches of a predefined length connected to a single node. The total number of branches was then summed and exported to Excel. To determine mean node and branch thickness, a Euclidean distance map was generated from the tube mask and “multiplied” by the node and skeletal branch masks respectively. Thickness values were calculated by summing the resulting pixels values in each of these images, multiplying these values by a factor of two and then by the pixel resolution, and lastly dividing by the total number of node pixels or skeletal branch pixels.

### Statistical analysis

Values were expressed as mean plus or minus standard deviations (SD). P values were based on the paired t-test. All the experiments were repeated at least three or more times. Results were considered statically significant with P value less than 0.05.

## Supporting Information

Figure S1 **Sequential connectivity map for VpepB. Figure is produced by CCPN Analysis 2.1.1.**
(JPG)

Figure S2 **Uptake of β_3_CT derived peptides by EC. Peptides were labeled with fluorescein (green) at the N-termini and added to HUVEC growth media for the indicated times.** The plasma membranes were labeled with Dil (red) and nuclei stained with DAPI (blue). The cells were fixed and analyzed by confocal microscopy.
(JPG)

Figure S3 **bFGF induced signaling events are not affected by F747 or pY747 peptides.** HUVEC cells pretreated with peptides as indicated and stimulated with bFGF for 30 min. Total cell lysates were immuno-probed with anti phospho-erk antibodies.
(JPG)
