# Supplementary figures and images for: Integrin β3 Crosstalk with VEGFR Accommodating Tyrosine Phosphorylation as a Regulatory Switch
Source: PLoS One. 2012 Feb 17;7(2):e31071. doi: 10.1371/journal.pone.0031071 (PMC3281915; doi:10.1371/journal.pone.0031071)

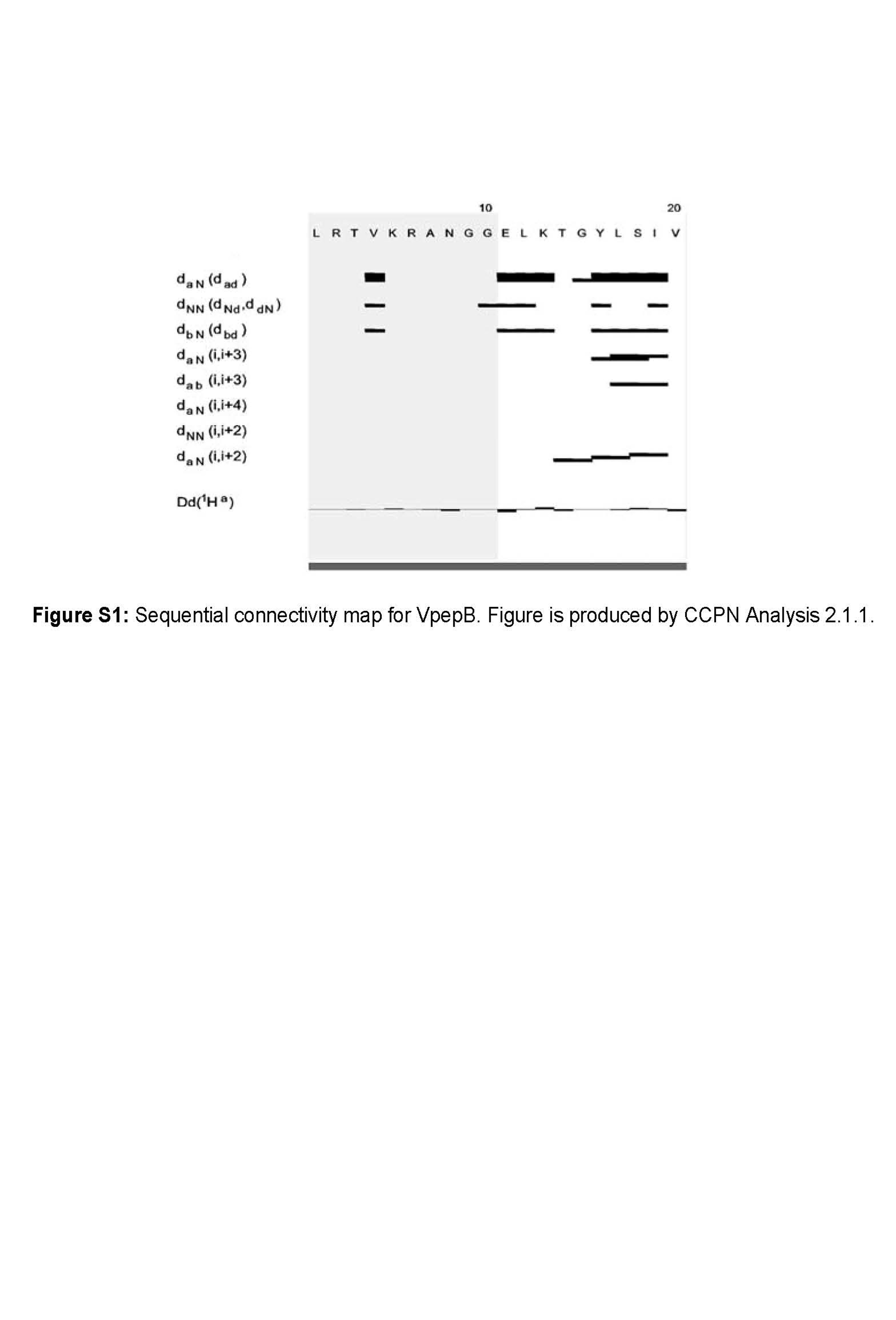

Supplement: Figure S1 — Sequential connectivity map for VpepB. Figure is produced by CCPN Analysis 2.1.1. (JPG) [file pone.0031071.s001.jpg]

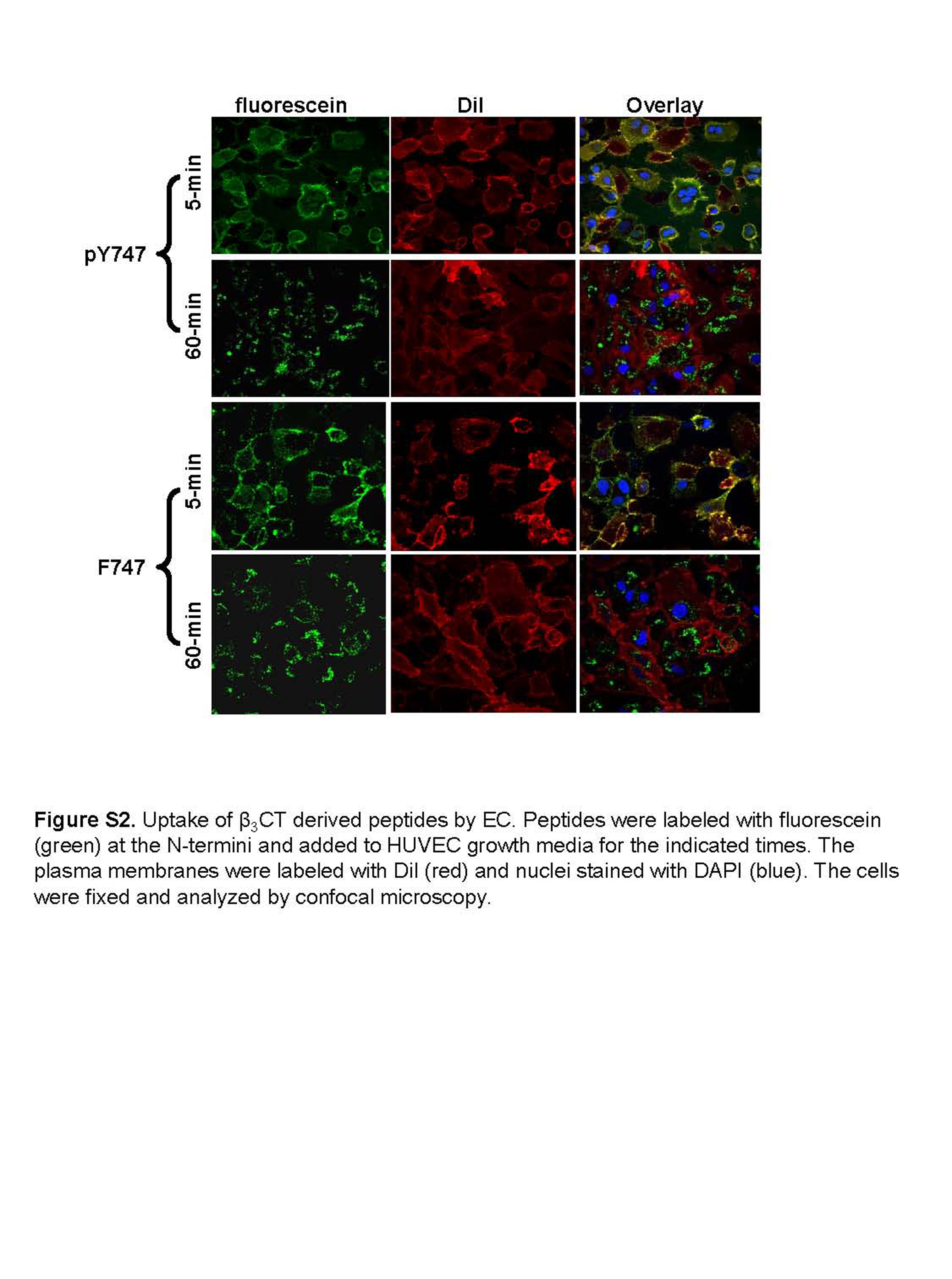

Supplement: Figure S2 — Uptake of β3CT derived peptides by EC. Peptides were labeled with fluorescein (green) at the N-termini and added to HUVEC growth media for the indicated times. The plasma membranes were labeled with Dil (red) and nuclei stained with DAPI (blue). The cells were fixed and analyzed by confocal microscopy. (JPG) [file pone.0031071.s002.jpg]

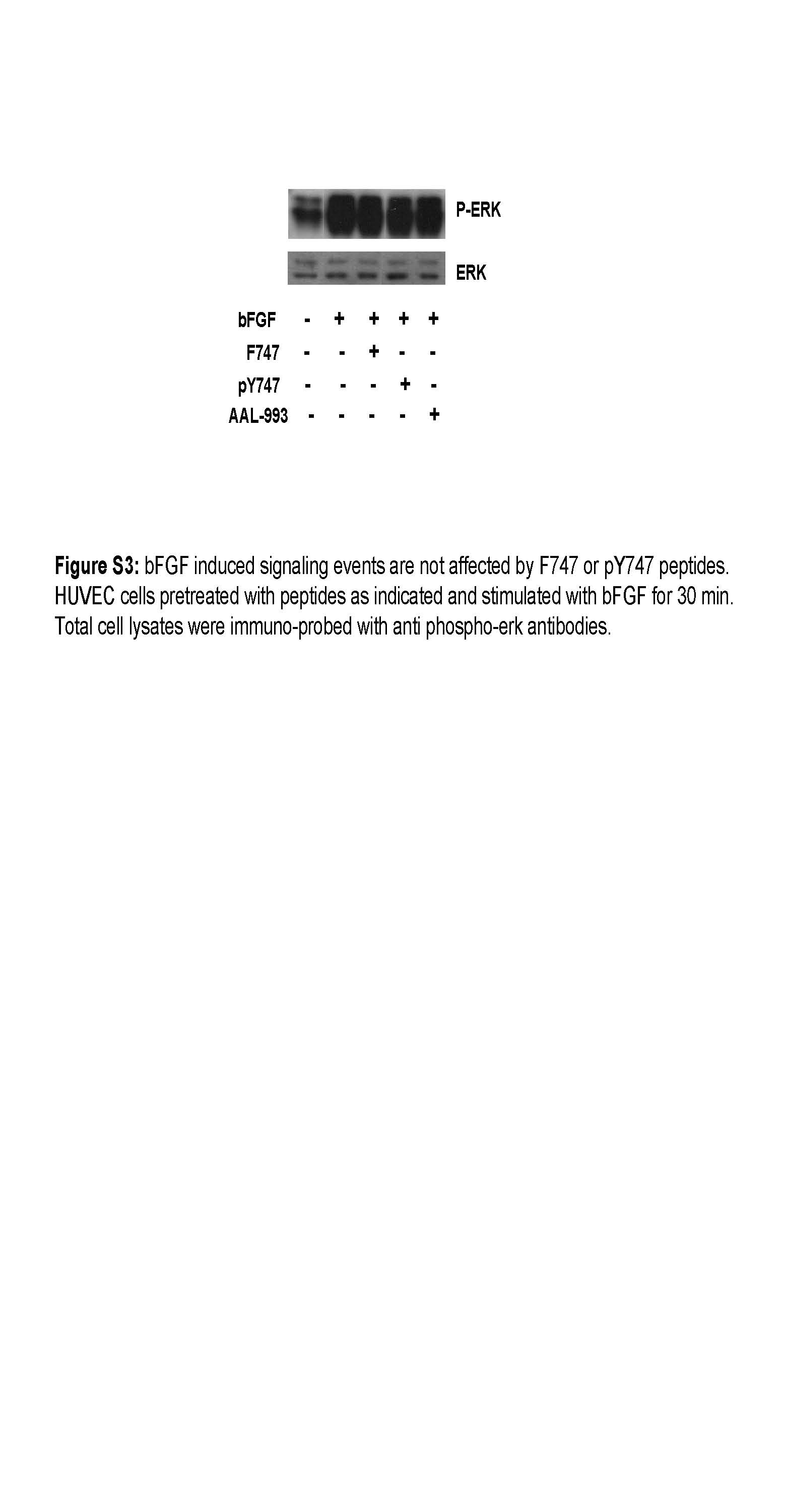

Supplement: Figure S3 — bFGF induced signaling events are not affected by F747 or pY747 peptides. HUVEC cells pretreated with peptides as indicated and stimulated with bFGF for 30 min. Total cell lysates were immuno-probed with anti phospho-erk antibodies. (JPG) [file pone.0031071.s003.jpg]
